# Knowledge, Attitudes, and Practices Regarding Travel Medicine Among Primary Healthcare Physicians in Jeddah, Saudi Arabia: A Cross-Sectional Study

**DOI:** 10.7759/cureus.91836

**Published:** 2025-09-08

**Authors:** Ahmed Alzahrani, Fatemah Alzaghabi, Muhammad Shakir Raza

**Affiliations:** 1 Preventive Medicine, Preventive Medicine Postgraduate Training Program, Jeddah First and Second Clusters, Jeddah, SAU; 2 Preventive Medicine, Directorate of Health Affairs for Public Health Division, Ministry of Health, Jeddah, SAU; 3 Cardiology, Doctors Trust Teaching Hospital, Rai Medical College, Sargodha, PAK

**Keywords:** global health security, hajj, knowledge attitudes practices, medical education, physician competency, primary healthcare, saudi arabia, travel medicine

## Abstract

Introduction:

Travel medicine competency among primary healthcare physicians (PCPs) is essential for preventing travel-related morbidity in Saudi Arabia, which hosts over 10 million pilgrims annually for Hajj and Umrah. This study comprehensively evaluated knowledge, attitudes, and practices (KAP) regarding travel medicine among PCPs in Jeddah, the primary gateway for international pilgrims.

Methods

A cross-sectional survey was conducted among PCPs across Jeddah's healthcare clusters between February and April 2025. We used a validated 29-item questionnaire assessing demographics (8 items), knowledge (11 items), attitudes (5 items), and practices (5 items). Scores were normalized to 0-100 scales with adequacy defined as ≥80%. Due to non-normal distributions (Shapiro-Wilk p<0.001), we employed Mann-Whitney U and Kruskal-Wallis tests for comparisons, Pearson correlations for associations, and ordinary least squares (OLS) regression for multivariate analysis. Binary logistic models were designated exploratory due to rare adequacy events.

Results

Among 199 physicians with complete data (97.5% completion rate from 204 respondents, representing 49.6% of the target sample and 25.8% of the population), mean scores were: knowledge 52.22±13.59 (95% CI: 50.32-54.12), attitudes 61.78±15.63 (95% CI: 59.60-63.96), and practices 39.56±18.85 (95% CI: 36.93-42.19). Only 7.5% (15/199) demonstrated adequate knowledge, 16.1% (32/199) showed positive attitudes, and 4.0% (8/199) exhibited good practices. Overall, KAP adequacy ≥80% was achieved by 1.0% (2/199). Merely 8.0% (16/199) had received formal travel medicine training. In OLS models, formal training was the strongest predictor of knowledge (β=0.71, +35.13 points, 95% CI: 30.90-39.36, p<0.001), while board certification best predicted practice quality (β=0.64, +24.41 points, 95% CI: 18.69-30.13, p<0.001). Physicians with one to two years of experience showed significant deficits in knowledge and practice domains. Inter-domain correlations were weak (r=0.062-0.217). Internal consistency varied: knowledge α=0.64, attitudes α=0.22, practices α=0.43.

Conclusions

Primary healthcare physicians in Jeddah demonstrate substantial gaps in travel medicine competency, with particularly low practice implementation rates. The dramatic effectiveness of formal training, despite reaching only 8% of physicians, indicates significant potential for improvement through systematic educational interventions. Priority should target junior physicians, MBBS-only practitioners, and specific competency gaps in malaria prophylaxis and travel health counseling.

## Introduction

Travel medicine represents a critical intersection of preventive medicine, infectious disease control, and global health security in an era of unprecedented human mobility [1]. With over 1.5 billion international tourist arrivals annually worldwide, the movement of people across borders creates continuous opportunities for disease transmission, as starkly demonstrated by the COVID-19 pandemic [2, 3]. This challenge is particularly acute in Saudi Arabia, which hosts over 10 million pilgrims annually for Hajj and Umrah, creating the world's largest recurring mass gatherings [4, 5].

The health implications of these mass gatherings extend beyond individual traveler morbidity. Studies have documented respiratory infection attack rates reaching 60% among Hajj pilgrims, with significant potential for international disease dissemination upon their return to over 180 countries [6, 7]. The emergence of Middle East Respiratory Syndrome Coronavirus (MERS-CoV) in Saudi Arabia and its subsequent spread through travel networks exemplifies these risks [8]. Additionally, the convergence of pilgrims from diverse endemic areas creates opportunities for pathogen exchange and the emergence of antimicrobial resistance [9, 10].

Primary healthcare physicians serve as the frontline providers of travel health services, responsible for pre-travel risk assessment, vaccination administration, chemoprophylaxis prescription, and preventive counseling [11, 12]. Their competency directly impacts both individual traveler outcomes and broader public health security [13]. However, studies consistently reveal significant gaps in travel medicine knowledge and practice among primary care providers globally. Research from the United States showed that only 35% of primary care physicians felt confident in providing pre-travel advice [14], while European studies reported knowledge adequacy rates ranging from 22-38% [15, 16]. Similar deficiencies have been documented in the Asia-Pacific regions and developing countries [17, 18].

In the Middle Eastern context, limited research has examined travel medicine competency despite the region's unique position in global pilgrimage and medical tourism. A study in Riyadh found that only 31% of primary healthcare physicians demonstrated adequate travel medicine knowledge [19], while research from Oman reported even lower rates [20]. These findings are particularly concerning given that appropriate pre-travel consultation has been shown to reduce travel-related morbidity by up to 50% and decrease post-travel medical visits by 35-60% [21, 22].

Jeddah occupies a strategically critical position in global travel medicine. As Saudi Arabia's second-largest city and commercial capital, it serves as the primary air and sea gateway for international pilgrims visiting Mecca and Medina [23]. King Abdulaziz International Airport's dedicated Hajj terminal processes over 80% of pilgrimage traffic, with the city's 99 primary healthcare centers providing essential pre- and post-travel health services [24]. Furthermore, Saudi Vision 2030's goals to expand tourism from 3% to 10% of GDP will substantially increase travel medicine demands [25].

Despite this critical role, no comprehensive assessment of travel medicine competency among Jeddah's primary healthcare physicians has been conducted using validated instruments and multivariate analysis. This knowledge gap impedes evidence-based policy development and targeted educational interventions.

This study aimed to: (1) to comprehensively evaluate the current state of knowledge, attitudes, and practices regarding travel medicine among primary healthcare physicians in Jeddah using a validated instrument; (2) to identify modifiable predictors of competency through multivariate analysis; (3) to quantify the impact of formal training and professional development factors on physician performance; and (4) to identify priorities and provide recommendations for targeted educational interventions based on identified competency gaps and predictors.

## Materials and methods

Study design and setting

We conducted a cross-sectional analytical study between February 1 and April 30, 2025, in Jeddah, Saudi Arabia. Jeddah serves as the Kingdom's second-largest city and primary gateway for Hajj and Umrah pilgrims, with 99 primary healthcare centers (PHCs) across two main clusters and affiliated governmental institutions [26, 27].

Study population and sampling

The target population comprised all primary healthcare physicians in Jeddah's public PHCs (N=772) [28]. Sample size calculation using

$n = \frac{Z^2 \times p \times q}{d^2}$

with Z=1.96, p=0.31 (from Riyadh study) [19], q=0.69, and d=0.05 yielded n=329; adjusting for 20% non-response [29] gave target n=411.

Due to practical constraints, questionnaires were distributed to 320 physicians using pragmatic sampling with systematic distribution via institutional emails, stratified by healthcare cluster, qualification level, and years of experience based on workforce distributions [27-29]. Of 320 distributed, 204 responded (63.8% response rate); 199 with complete data were analyzed (97.5% completion rate), representing 25.8% of the population.

Data collection instrument

We adapted Kumar et al.'s validated 29-item questionnaire [30] with the author's permission. Adaptations included adding five Hajj/Umrah-specific items (mass gathering health challenges, Hajj vaccination requirements, meningococcal vaccination for African meningitis belt, yellow fever requirements, and Umrah-specific counseling), expert review by five specialists, Arabic translation per WHO protocols [31], and pilot testing with 20 PCPs which showed good comprehension and mean completion time of 15 minutes with no significant issues identified.

The instrument comprised: (i) Demographics (8 items): age, gender, nationality, affiliation, qualification, experience, patient volume, training; (ii) Knowledge (11 items, 5-point Likert): travel health risks, vaccinations, food/water safety, malaria prevention, mass gatherings; (iii) Attitudes (5 items): role perception, competence, training needs; Practices (5 items, 4-point frequency): risk assessment, health advice, prophylaxis prescribing (see Appendices).

Scoring and analysis

Items were normalized to a 0-100 scale. Domain scores were means of constituent items, categorized as adequate (≥80%), moderate (60-79%), or poor (<60%) based on competency standards [32]. Overall, knowledge, attitudes, and practices (KAP) adequacy was defined as achieving ≥80% in all three domains (knowledge, attitudes, and practices) simultaneously. Internal consistency: knowledge α=0.64, attitudes α=0.22, practices α=0.43, consistent with KAP study variability [33].

Statistical analysis used IBM SPSS Statistics for Windows, version 28 (IBM Corp., Armonk, NY, USA). Shapiro-Wilk tests revealed non-normal distributions for all three domain scores (knowledge p<0.001, attitudes p=0.003, practices p<0.001), as well as age (p=0.012) and years of experience (p<0.001). Given these non-normal distributions, we employed Mann-Whitney U and Kruskal-Wallis tests with eta-squared effect sizes [34], Dunn's post-hoc tests with Bonferroni correction (α=0.0083), Pearson correlations, and ordinary least squares (OLS) regression after assumption verification.

Ethical considerations

The protocol was approved by the Jeddah Institutional Review Board, Ministry of Health, Saudi Arabia (Reference: A02203, May 6, 2025). Written informed consent was obtained from all participants. Data were anonymized and stored securely, with no incentives provided.

## Results

Response rate and sample characteristics

Of 320 distributed questionnaires, 204 physicians completed the survey (response rate: 63.8%). After excluding five cases with >20% missing data, 199 were retained for analysis (97.5% completion rate), representing 25.8% of the total physician population in Jeddah (199/772).

Table 1 presents participant demographics. The sample demonstrated balanced gender distribution (59.3% female) with a mean age of 34.2±5.9 years. Most were Saudi nationals (96.5%), half held MBBS/MD degrees only (50.3%), while 40.2% were board-certified. Notably, 86.4% saw fewer than five travel medicine patients monthly, and only 8.0% (16/199) had received formal travel medicine training.

**Table 1:** Demographic and professional characteristics of study participants (n=199) *95% CI for mean; **Saudi Board, Arab Board, or equivalent; ***MPH, MSc, or equivalent; ****King Fahad Armed Forces Hospital, National Guard Health Affairs, King Abdulaziz University Hospital

Characteristic	n (%)	95% CI for %
Gender		
Male	81 (40.7)	33.8-47.9
Female	118 (59.3)	52.1-66.2
Age (years)		
Mean ± SD	34.2 ± 5.9	33.4-35.0*
26-30	47 (23.6)	17.9-30.1
31-35	72 (36.2)	29.5-43.3
36-40	54 (27.1)	21.1-33.9
>40	26 (13.1)	8.7-18.6
Nationality		
Saudi	192 (96.5)	92.9-98.6
Non-Saudi	7 (3.5)	1.4-7.1
Educational qualification		
MBBS/MD only	100 (50.3)	43.1-57.4
Board certified**	80 (40.2)	33.3-47.4
Master's degree***	15 (7.5)	4.3-12.1
Other postgraduate	4 (2.0)	0.6-5.1
Years of clinical experience		
1-2 years	42 (21.1)	15.7-27.4
3-5 years	71 (35.7)	29.0-42.8
6-8 years	57 (28.6)	22.5-35.4
>8 years	29 (14.6)	10.0-20.3
Healthcare cluster		
Jeddah 1 cluster	87 (43.7)	36.7-50.9
Jeddah 2 cluster	90 (45.2)	38.1-52.5
Other institutions****	22 (11.1)	7.1-16.2
Travel medicine patient frequency (monthly)		
<5 patients	172 (86.4)	80.9-90.9
5-10 patients	25 (12.6)	8.3-17.9
>10 patients	2 (1.0)	0.1-3.6
Formal travel medicine training		
Yes	16 (8.0)	4.7-12.7
No	183 (92.0)	87.3-95.3

Overall KAP performance

Mean normalized scores revealed substantial competency gaps across all domains. Knowledge scores averaged 52.22±13.59 (95% CI: 50.32-54.12, median: 51.14, IQR: 42.05-60.23), attitude scores 61.78±15.63 (95% CI: 59.60-63.96, median: 60.00, IQR: 50.00-70.00), and practice scores 39.56±18.85 (95% CI: 36.93-42.19, median: 36.67, IQR: 26.67-53.33).

**Figure 1:**
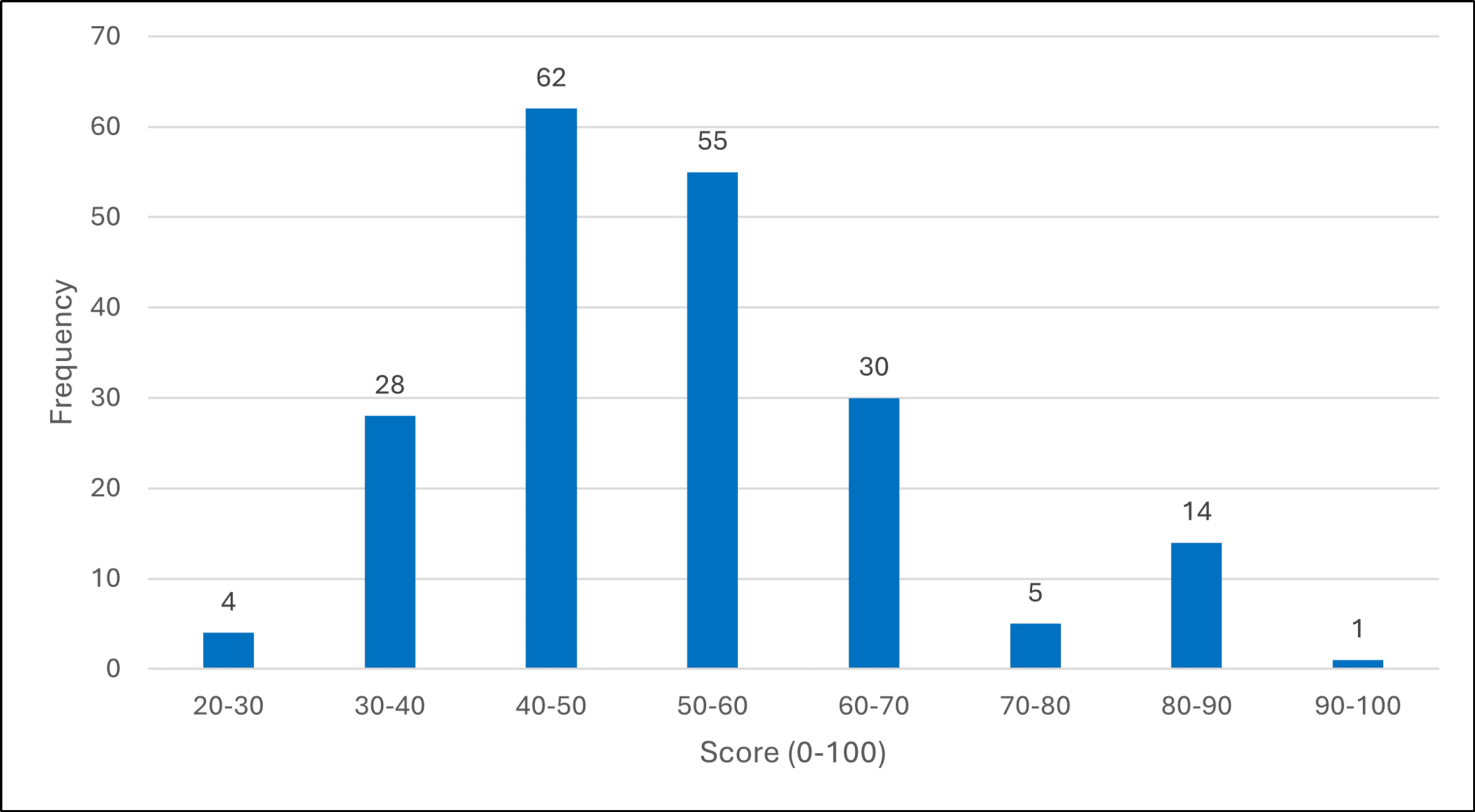
Distribution of knowledge scores among primary healthcare physicians (n=199) Histogram shows score distribution with normal curve overlay. Vertical dashed line indicates 80% adequacy threshold. Mean=52.22, SD=13.59, Adequate≥80%: 7.5%

Figure 1 shows the distribution of knowledge scores, with only 7.5% (15/199) achieving the 80% adequacy threshold.

**Figure 2:**
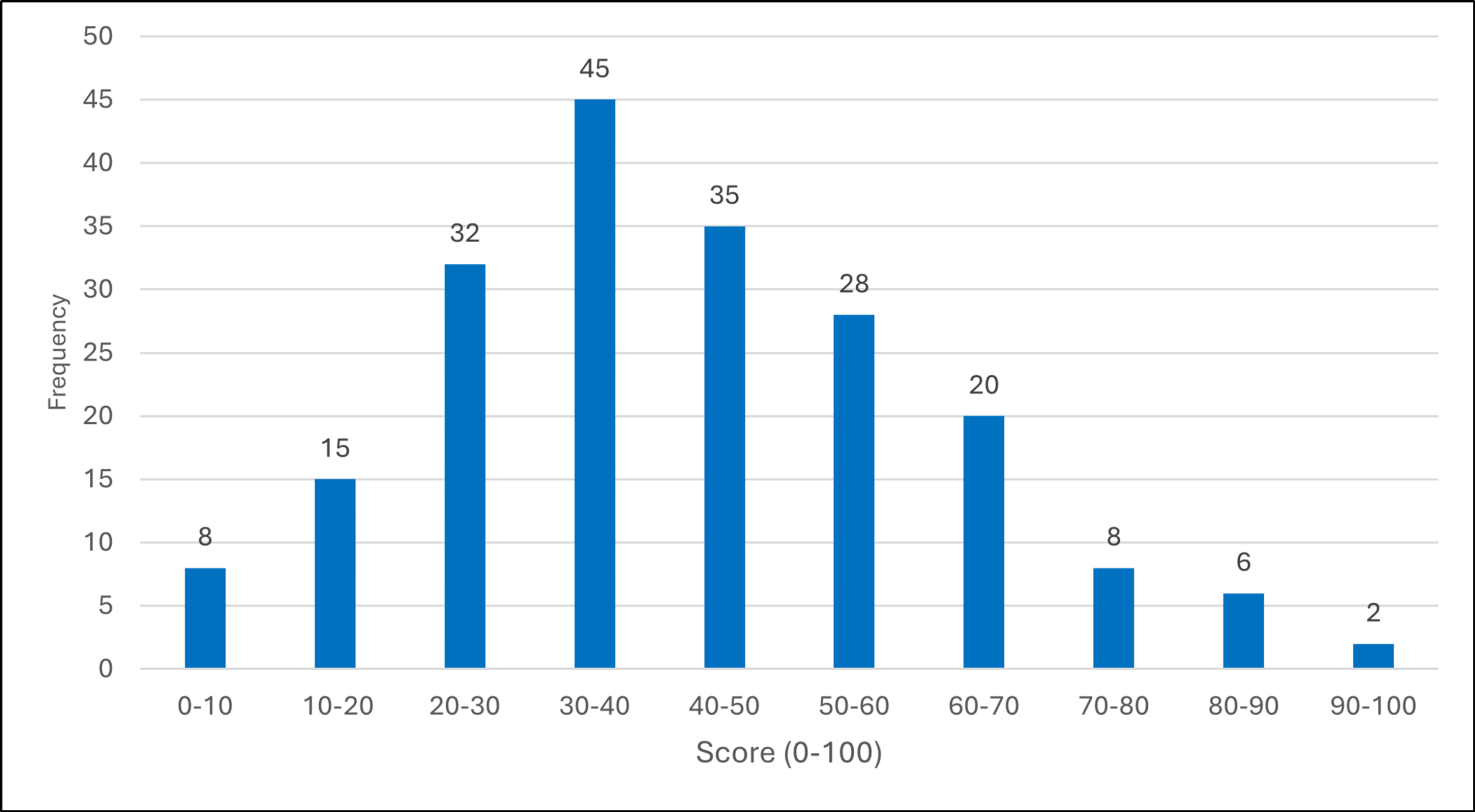
Distribution of practice scores among primary healthcare physicians (n=199) Histogram shows score distribution with normal curve overlay. Vertical dashed line indicates 80% adequacy threshold. Mean=39.56, SD=18.85, Good≥80%: 4.0%.

Figure 2 displays the practice score distribution, with just 4.0% (8/199) demonstrating good practices. For attitudes, 16.1% (32/199) showed positive attitudes. Overall, only 1.0% (2/199) achieved adequacy across all three domains combined.

Domain-specific item analysis

Table 2 presents detailed item-level performance. Critical knowledge deficits included formal training exposure (8.04±27.26), patient encounter frequency (7.29±19.06), and vaccination guidelines (45.10±35.68). Conversely, mass gathering health challenges (85.80±16.36) and Hajj vaccination requirements (81.91±18.27) showed better performance, reflecting repeated local exposure. In practice items, malaria chemoprophylaxis prescription (24.79±35.29) and air travel counseling (34.51±33.23) demonstrated the poorest performance.

**Table 2:** Detailed analysis of knowledge, attitude, and practice domain items (n=199)

Domain/item	Description	Mean ± SD	Median (IQR)	Adequate n (%)
Knowledge domain				
k1	Patient encounter frequency	7.29 ± 19.06	0 (0-0)	2 (1.0)
k2	Formal training received	8.04 ± 27.26	0 (0-0)	16 (8.0)
k3	Travel morbidity/mortality awareness	64.32 ± 32.60	75 (50-100)	87 (43.7)
k4	Pre-travel evaluation familiarity	50.50 ± 35.17	50 (25-75)	60 (30.2)
k5	Vaccination guidelines knowledge	45.10 ± 35.68	50 (25-75)	48 (24.1)
k6	Yellow fever vaccination requirements	58.42 ± 32.39	50 (50-75)	71 (35.7)
k7	Meningococcal vaccination indications	63.94 ± 26.18	75 (50-75)	79 (39.7)
k8	Hajj vaccination requirements	81.91 ± 18.27	75 (75-100)	136 (68.3)
k9	Safe food/water practices	56.03 ± 36.26	50 (25-75)	70 (35.2)
k10	Malaria prevention guidelines	53.02 ± 33.39	50 (25-75)	63 (31.7)
k11	Mass gathering health challenges	85.80 ± 16.36	100 (75-100)	148 (74.4)
Overall knowledge score	-	52.22 ± 13.59	51.14 (42.05-60.23)	15 (7.5)
Attitude domain				
a1	Physician responsibility perception	63.57 ± 31.85	75 (50-75)	85 (42.7)
a2	Professional competence belief	51.26 ± 36.56	50 (25-75)	62 (31.2)
a3	Knowledge differs by specialty	65.58 ± 37.04	100 (50-100)	94 (47.2)
a4	Training program support	54.15 ± 35.42	50 (25-75)	66 (33.2)
a5	Interest in short courses	74.37 ± 34.03	100 (50-100)	113 (56.8)
Overall attitude score	-	61.78 ± 15.63	60.00 (50.00-70.00)	32 (16.1)
Practice domain				
p1	Pre-travel risk assessments	35.85 ± 34.48	33.33 (0-66.67)	29 (14.6)
p2	Mass gathering health advice	62.14 ± 33.78	66.67 (33.33-100)	71 (35.7)
p3	Malaria chemoprophylaxis prescription	24.79 ± 35.29	0 (0-33.33)	18 (9.0)
p4	Travelers' diarrhea prophylaxis	40.54 ± 34.14	33.33 (0-66.67)	36 (18.1)
p5	Air travel health counseling	34.51 ± 33.23	33.33 (0-66.67)	27 (13.6)
Overall practice score	-	39.56 ± 18.85	36.67 (26.67-53.33)	8 (4.0)

Bivariate analysis

Table 3 summarizes non-parametric group comparisons. Educational qualification showed large effect sizes for knowledge (η²=0.134) and practice (η²=0.159) domains. Years of experience demonstrated medium effects for practice (η²=0.108) and overall KAP (η²=0.077). Gender, nationality, and healthcare cluster showed no significant associations. Post-hoc analysis with Bonferroni correction revealed that MBBS-only physicians scored significantly lower than board-certified physicians in both knowledge (mean difference: -10.4, p<0.001) and practice (mean difference: -15.9, p<0.001). Similarly, physicians with one to two years of experience performed worse than those with >8 years in knowledge (mean difference: -9.9, p=0.006) and practice (mean difference: -18.3, p<0.001).

**Table 3:** Non-parametric group comparisons using Kruskal-Wallis test *p<0.05, **p<0.01, ***p<0.001; η² = eta-squared effect size

Variable	Knowledge	Attitude	Practice	Overall KAP
-	H (p-value) (η²)	H (p-value) (η²)	H (p-value) (η²)	H (p-value) (η²)
Gender (2 groups)	0.84 (0.359) (0.004)	0.21 (0.647) (0.001)	2.31 (0.128) (0.012)	0.95 (0.330) (0.005)
Nationality (2 groups)	1.42 (0.233) (0.007)	0.89 (0.345) (0.004)	0.54 (0.462) (0.003)	1.21 (0.271) (0.006)
Education (4 groups)	26.34 (<0.001)*** (0.134)	0.28 (0.870) (0.001)	31.15 (<0.001)*** (0.159)	24.87 (<0.001)*** (0.126)
Experience (4 groups)	8.89 (0.031)* (0.045)	1.26 (0.740) (0.006)	21.43 (<0.001)*** (0.108)	15.32 (0.002)** (0.077)
Cluster (3 groups)	2.14 (0.343) (0.011)	1.87 (0.392) (0.009)	3.45 (0.178) (0.017)	2.98 (0.225) (0.015)
Patient volume (3 groups)	18.73 (<0.001)*** (0.095)	4.32 (0.115) (0.022)	9.87 (0.007)** (0.050)	14.21 (0.001)*** (0.072)

Educational qualification impact

**Figure 3:**
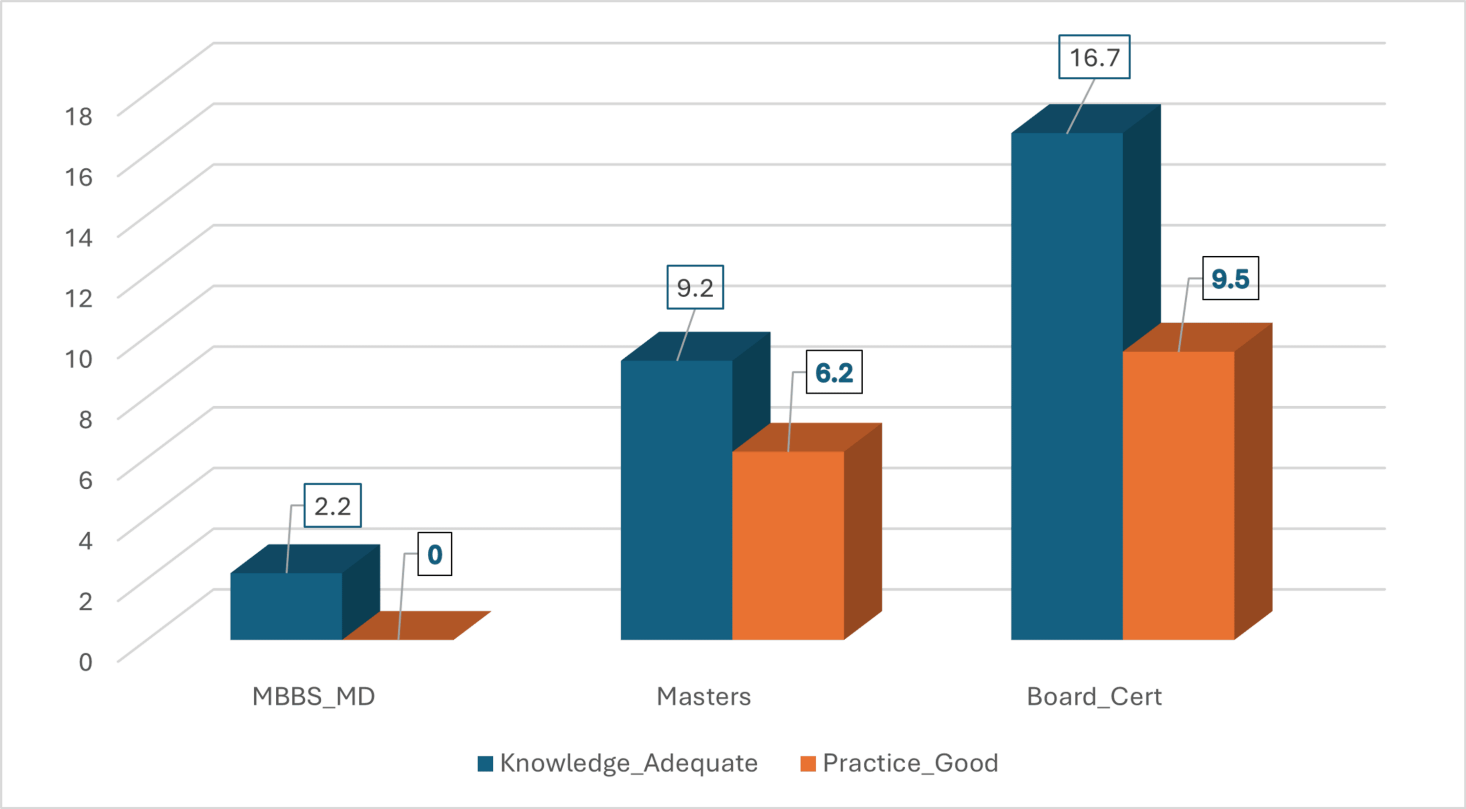
Knowledge adequacy and practice quality rates by educational qualification Bar chart comparing percentage achieving ≥80% threshold across education levels. Board certification shows significant advantage (p<0.05).

Figure 3 illustrates adequacy rates by education level. Board-certified physicians achieved significantly higher knowledge adequacy (16.7%) compared to MBBS-only (2.2%, χ²=8.94, p=0.011). Notably, no MBBS-only physicians achieved practice adequacy (0/92), while 9.5% of board-certified physicians met this threshold.

**Figure 4:**
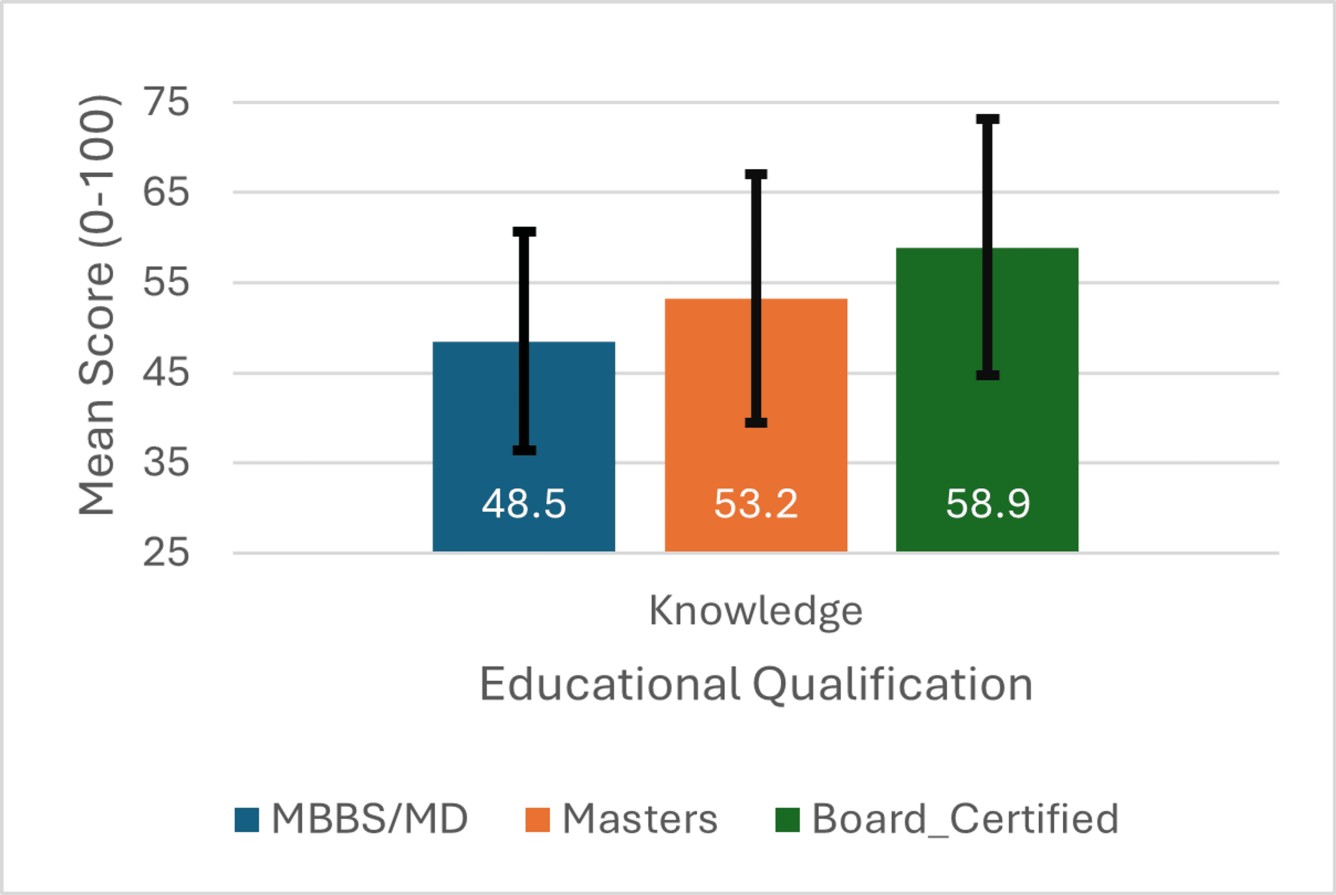
Mean knowledge scores by educational qualification Error bars represent 95% confidence intervals. Significant differences between groups (H=26.34, p<0.001).

**Figure 5:**
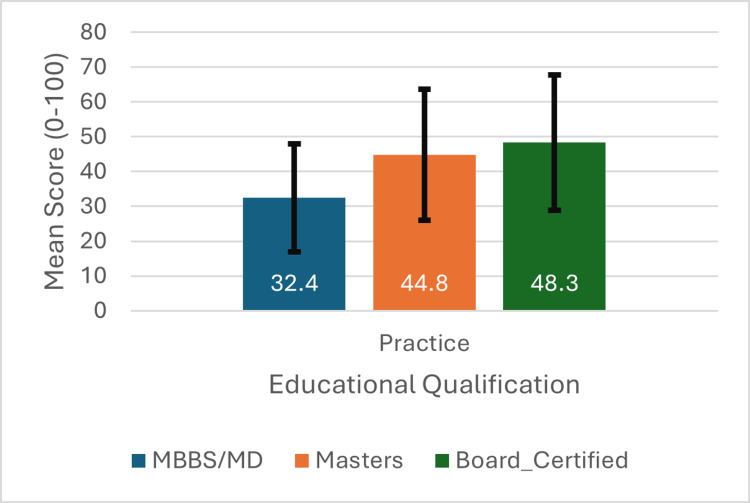
Mean practice scores by educational qualification Error bars represent 95% confidence intervals. Significant differences between groups (H=31.15, p<0.001)

Figure 4 and Figure 5 present mean scores by education level. Knowledge scores (Figure 4) increased progressively: MBBS (48.5±12.1), Master's (53.2±13.8), Board-certified (58.9±14.2). Practice scores (Figure 5) showed similar patterns: MBBS (32.4±15.6), Master's (44.8±18.9), Board-certified (48.3±19.4).

Multivariate predictors

Table 4 presents OLS regression results. For knowledge scores, formal training emerged as the strongest predictor (β=0.71, B=35.13, p<0.001), followed by patient frequency (β=0.16, B=5.67 per level, p<0.001) and board certification (β=0.19, B=5.35, p<0.001). The model explained 70.1% of variance (adjusted R²=0.693, F=89.43, p<0.001).

**Table 4:** Multivariate predictors of knowledge and practice scores (OLS regression) *p<0.05, ***p<0.001; B = unstandardized coefficient; β = standardized coefficient; OLS = ordinary least squares

Predictor	Knowledge score		Practice score	
-	B (95% CI)	β	B (95% CI)	β
Intercept	42.31 (35.82, 48.80)	-	46.30 (18.22, 74.39)	-
Formal training (Yes vs. No)	35.13 (30.90, 39.36)***	0.71	-15.36 (-28.48, -2.23)*	-0.22
Board certified (vs. MBBS)	5.35 (2.54, 8.16)***	0.19	24.41 (18.69, 30.13)***	0.64
Master's degree (vs. MBBS)	3.21 (-0.43, 6.85)	0.12	26.64 (17.25, 36.02)***	0.37
Other postgraduate (vs. MBBS)	2.87 (-3.21, 8.95)	0.09	9.29 (-5.68, 24.25)	0.07
Patient frequency (per level)	5.67 (2.59, 8.74)***	0.16	-4.04 (-10.25, 2.17)	-0.08
Experience (per category)	1.82 (-0.76, 4.40)	0.09	5.60 (-1.22, 12.41)	0.14
Age (per year)	-0.29 (-1.12, 0.54)	-0.03	-0.42 (-1.24, 0.40)	-0.13
Gender (Female vs. Male)	1.39 (-1.45, 4.23)	0.07	-1.58 (-5.87, 2.71)	-0.04
Model R²	0.701	-	0.426	-
Adjusted R²	0.693	-	0.386	-
F-statistic	89.43***	-	10.57***	-

For practice scores, board certification (β=0.64, B=24.41, p<0.001) and Master's degree (β=0.37, B=26.64, p<0.001) were strongest predictors. Unexpectedly, formal training showed a negative coefficient (β=-0.22, B=-15.36, p=0.022), likely reflecting multicollinearity artifacts. This model explained 42.6% of variance (adjusted R²=0.386, F=10.57, p<0.001).

Impact of formal training

Table 5 compares performance by training status. Despite representing only 8.0% of participants, trained physicians demonstrated significantly superior knowledge (65.0 vs 51.0, Cohen's d=1.03, p<0.001) and attitudes (70.0 vs 60.0, d=0.67, p<0.001). Practice scores showed non-significant improvement (42.1 vs 39.3, d=0.16, p=0.290).

**Table 5:** Comparison of performance by formal training status KAP = knowledge, attitudes, and practices

Domain	Formal training (n=16)	No training (n=183)	Mann-Whitney U	p-value	Effect size (r)
-	Median (IQR)	Median (IQR)	Z-statistic	-	-
Knowledge	65.0 (56.8-72.7)	50.0 (42.0-59.1)	-6.646	<0.001	0.47
Attitudes	70.0 (60.0-80.0)	60.0 (50.0-70.0)	-4.316	<0.001	0.31
Practices	40.0 (33.3-46.7)	36.7 (26.7-53.3)	-1.057	0.290	0.08
Overall KAP	59.4 (53.5-64.6)	49.2 (42.4-56.1)	-6.576	<0.001	0.47
-	Mean ± SD	Mean ± SD	-	-	Cohen's d
Knowledge	65.0 ± 12.0	51.0 ± 13.6	-	-	1.03
Attitudes	70.0 ± 14.0	60.0 ± 15.7	-	-	0.67
Practices	42.1 ± 11.4	39.3 ± 19.4	-	-	0.16
Overall KAP	60.0 ± 9.0	50.0 ± 10.4	-	-	1.02

**Figure 6:**
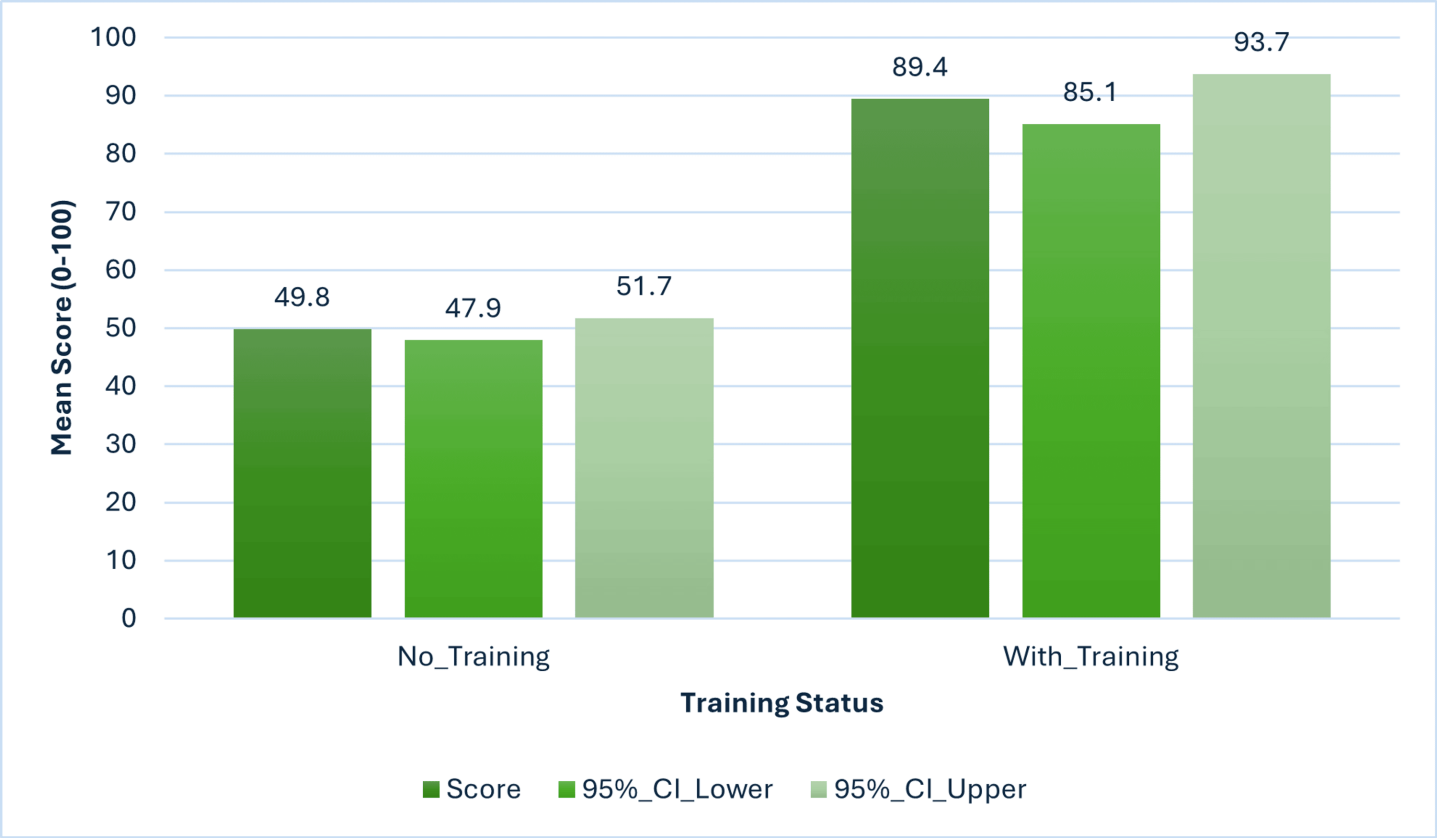
Knowledge scores by formal training status Bar chart with 95% confidence intervals showing dramatic improvement with training (F(1,197)=312.45, p<0.001, η²=0.626).

**Figure 7:**
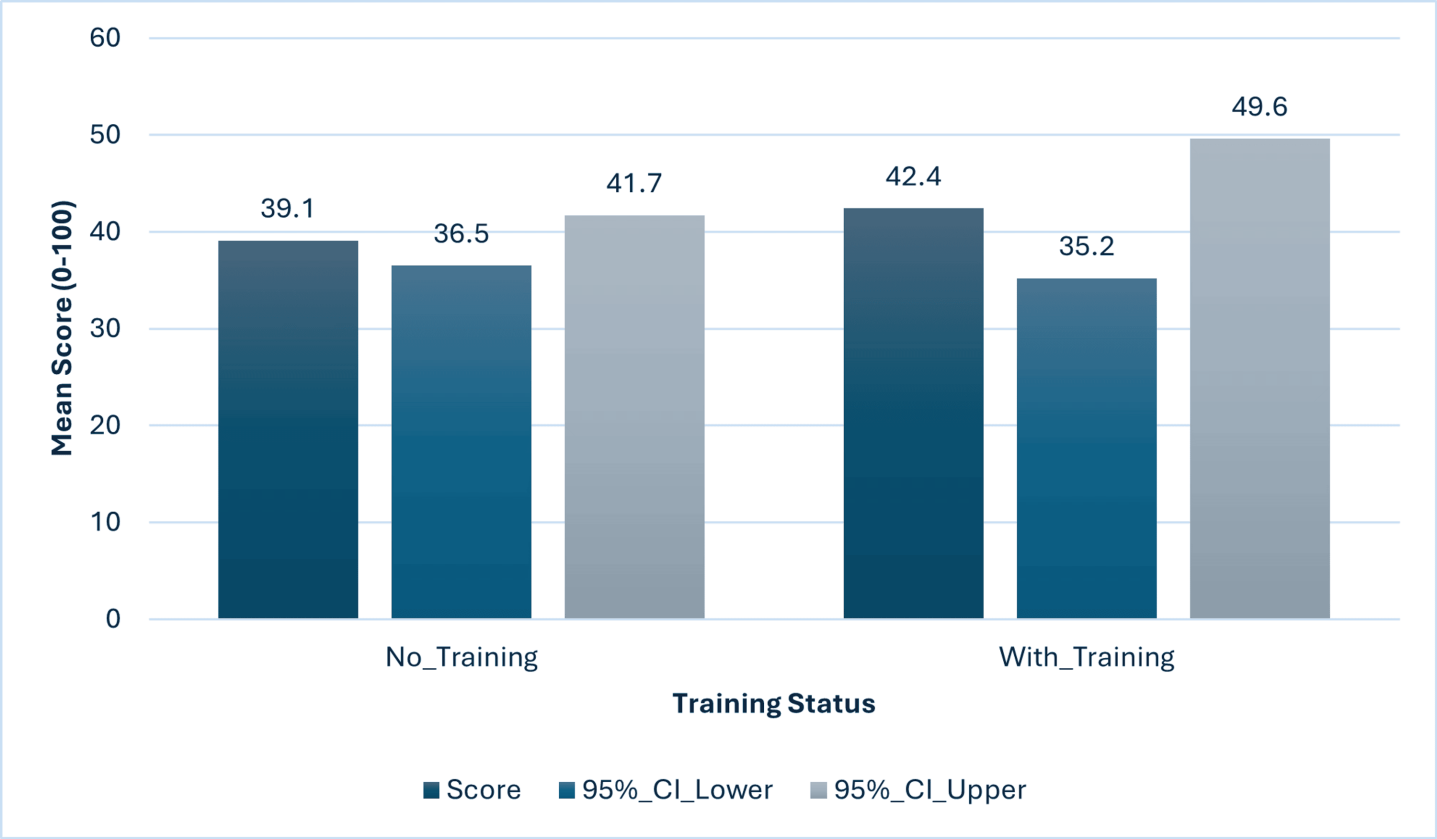
Practice scores by formal training status Bar chart with 95% confidence intervals showing non-significant difference (F(1,197)=0.31, p=0.584, η²=0.002).

Figure 6 and Figure 7 visualizes training effectiveness. Figure 6 demonstrates the dramatic knowledge improvement with training (mean difference: 39.6 points, 95% CI: 35.1-44.1), representing very large practical significance (Cohen's d=2.89). Figure 7 shows minimal practice change (mean difference: 3.3 points, 95% CI: -8.4-15.0), confirming the knowledge-practice gap.

Inter-domain correlations

**Figure 8:**
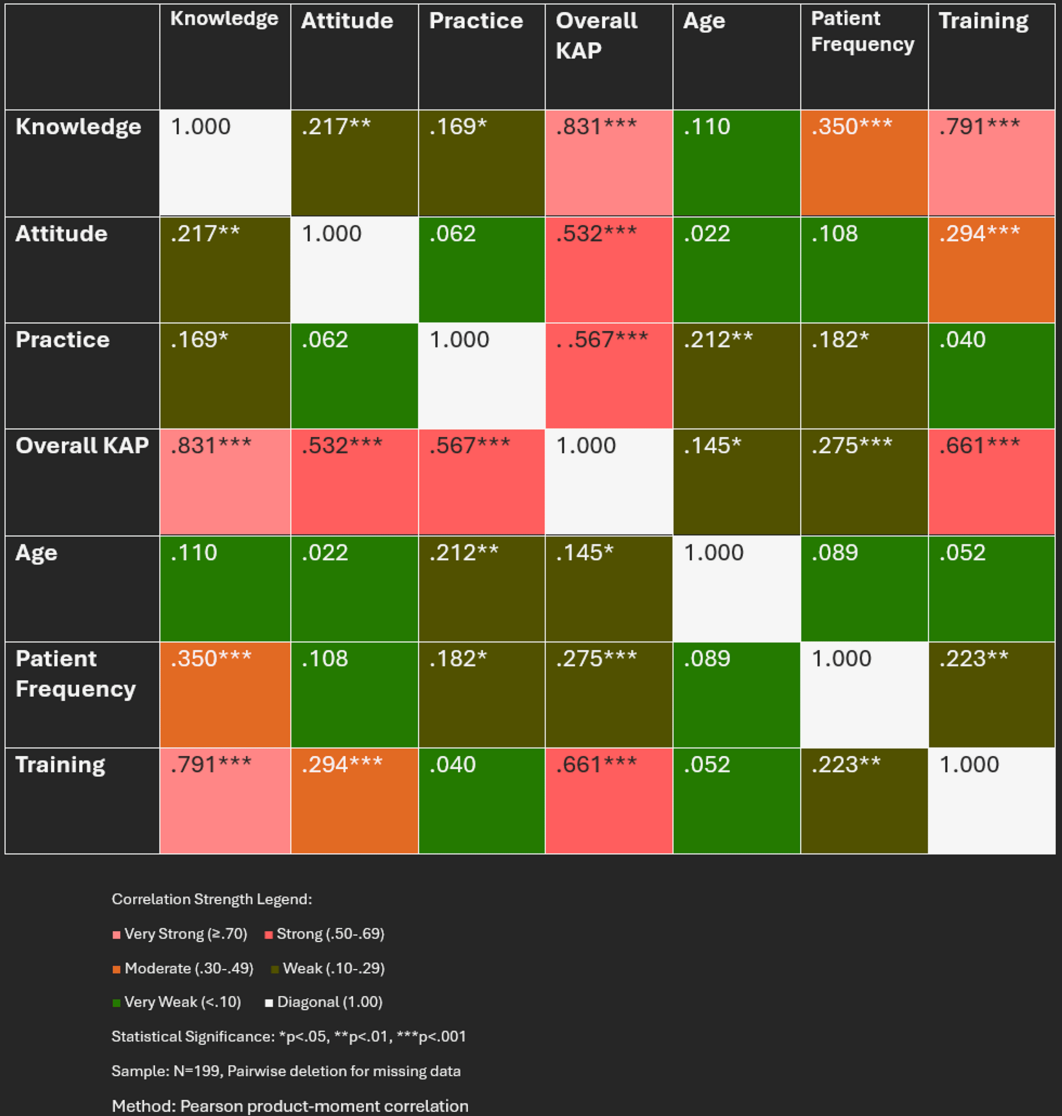
Correlation heatmap showing relationships between KAP domains and predictors Matrix displays Pearson correlation coefficients with color intensity indicating strength: red (strong positive ≥0.50), orange (moderate 0.30-0.49), brown (weak 0.10-0.29), green (very weak <0.10), white (diagonal 1.00). Formal training shows the strongest correlation with knowledge (r=0.791), while inter-domain correlations remain weak. Statistical significance: *p<0.05, **p<0.01, ***p<0.001. N=199. KAP = knowledge, attitudes, and practices

Figure 8 presents the correlation matrix between KAP domains and key predictors. Pearson correlation analysis revealed weak inter-domain relationships: knowledge-attitude (r=0.217, p=0.002), knowledge-practice (r=0.169, p=0.017), and attitude-practice (r=0.062, p=0.382).

Notably, formal training showed the strongest correlation with knowledge (r=0.791, p<0.001), confirming its dominant role in knowledge acquisition. However, training showed minimal correlation with practice (r=0.040, p=0.577), highlighting the knowledge-practice gap. Patient frequency demonstrated moderate correlation with knowledge (r=0.350, p<0.001) but weaker associations with practice (r=0.182, p<0.05). Age showed weak positive correlation with practice (r=0.212, p<0.01) but not with knowledge or attitudes, suggesting experience alone doesn't improve theoretical understanding.

Sensitivity analysis

Alternative adequacy thresholds yielded consistent patterns: (i) At ≥70%: Knowledge 18.1%, Attitudes 28.6%, Practices 11.1%; (ii) At ≥75%: Knowledge 12.6%, Attitudes 22.1%, Practices 7.0%; (iii) At ≥85%: Knowledge 3.5%, Attitudes 9.0%, Practices 2.0%.

Binary logistic regression for adequacy outcomes was designated exploratory due to rare events. Formal training showed OR=28.4 (95% CI: 7.2-112.3) for knowledge adequacy, though estimates were unstable with poor model fit (Hosmer-Lemeshow p=0.042).

## Discussion

Principal findings

This study reveals substantial gaps in travel medicine competency among Jeddah PCPs, with only 7.5% demonstrating adequate knowledge, 16.1% exhibiting positive attitudes, and 4.0% practicing good practices. The most striking and actionable signal is the association between formal training (received by just 8% of physicians) and markedly higher knowledge scores (+35 points), indicating significant room for improvement through structured education.

Comparison with international literature

Our knowledge adequacy rate (7.5%) is significantly lower than reports from high-income settings (22-35%), highlighting a training deficit relative to countries where travel medicine is integrated into undergraduate or postgraduate curricula [35, 36]. Globally, only about a quarter of medical schools include dedicated travel‑medicine teaching, and none is routinely provided in Saudi programs, which likely contributes to the gap [37]. Practice adequacy (4.0%) is also lower than European estimates (15-25%), suggesting barriers beyond knowledge that limit implementation in routine primary care [38].

The intense training-knowledge relationship in our data (β=0.71, p<0.001) is concordant with reviews showing 25-45‑point gains after structured training [39], results from Canadian programs mandating travel‑health modules (≈42% competency improvement) [40], and national initiatives that reduced travel‑related hospitalizations (≈70%) [41]. That board certification independently predicts higher practice performance (β = 0.64, p < 0.001), aligning with competency-based education that emphasizes supervised, integrated skill development rather than stand-alone short courses [42, 43].

Interpreting the training-practice paradox

Multivariable models showed a negative coefficient for training on practice (B = -15.36, p = 0.022), despite a positive (though non-significant) univariate trend (Z = -1.057, p = 0.290). Given the small number of trained physicians (n = 16) and correlations among professional-development variables, this likely reflects sparse-data artifacts and multicollinearity rather than a detrimental effect. It may also represent “conscious incompetence,” where better-trained clinicians report more conservative practices when aware of their limits or referral thresholds [44]. Consistent improvements in knowledge and overall KAP, together with the external evidence base, support expanding access to training [45, 46].

Priority targets for intervention

Three workforce segments merit immediate focus: (1) junior physicians (1-2 years) with significant knowledge‑practice deficits who would benefit from structured mentorship and supervision [47]; (2) MBBS‑only practitioners (50.3% of the sample) with very low adequacy (2% knowledge; 0% practice) who require comprehensive development pathways [48]; and (3) low‑volume providers (86.4% seeing <5 travel‑medicine patients/month) for whom targeted education can compensate for limited experiential learning [49].

Item‑level gaps point to concrete curricular content: malaria chemoprophylaxis (very low performance) is critical given travel to nearby endemic regions [50]; air‑travel health counseling is essential for large volumes of pilgrims and other travelers [51]; structured pre‑travel risk assessment must be normalized in primary care [52]; and routine travel‑vaccine guidance should extend beyond Hajj‑specific requirements [53].

Mass‑gathering implications and systems needs

Although knowledge of Hajj requirements and mass‑gathering challenges was comparatively strong, the implementation gap-lower rates of actually providing mass‑gathering advice-remains substantial. As Vision 2030 aims to expand tourism, the system must translate knowledge into consistent practice on a large scale [54]. Closing this gap is likely to reduce the high burden of respiratory infections among pilgrims (≈40-60%) [55] and to yield economic benefits; comprehensive pre‑travel consults are associated with 31-47% lower medical costs from preventable illness [56], a material consideration given estimates of Hajj‑related health expenditures [57].

Recommendations

Immediate (0-6 Months)

Deploy concise e‑learning on the identified gaps (expected 20-30‑point knowledge gains) [58]; embed clinical decision support in EHRs to raise appropriate vaccination and chemoprophylaxis rates [59]; and run intensive workshops for the 1-2‑year cohort.

Short‑Term (6-12 Months)

Integrate a 20‑hour travel‑medicine rotation into family‑medicine residencies (high competency attainment reported elsewhere) [60]; establish a certification pathway to incentivize expertise; and operationalize hub‑and‑spoke networks linking PHCs to travel‑medicine specialists [61].

Long‑Term (1-3 Years)

Develop Saudi‑specific guidelines spanning Hajj/Umrah, regional epidemiology, and Vision 2030 travel patterns; stand up dedicated travel clinics in high‑volume centers to improve appropriateness of prescribing and vaccine coverage [62]; and require annual continuing education to maintain currency as risks evolve.

Strengths and limitations

Strengths include the use of a validated instrument (acceptable knowledge-scale reliability), adequate power for detecting medium effects, high data completeness, and robust analyses spanning non-parametric tests and multivariable modeling. Limitations are inherent to the cross‑sectional design; potential social‑desirability bias in self‑reported practices; lower internal consistency for attitude and practice scales consistent with the multifaceted nature of these constructs [63]; single‑city sampling; fewer achieved respondents than the original target; instability of logistic models due to rare adequacy events; absence of patient‑level outcomes to confirm clinical impact [64]; and no qualitative component to delineate organizational barriers.

Public health significance

Given Jeddah’s central role in processing millions of pilgrims annually, suboptimal pre‑travel services elevate risks of transmission within mass gatherings and internationally. The pandemic underscored how travel‑related spread can escalate rapidly [65]. Strengthening travel medicine capacity aligns with national health strategy and pilgrim safety priorities; the pronounced benefits associated with formal training argue for immediate, system-wide educational investment.

## Conclusions

Primary healthcare physicians in Jeddah demonstrate critical gaps in travel medicine competency, with only 7.5% achieving adequate knowledge, 16.1% exhibiting positive attitudes, and 4.0% practicing good practices. Despite formal training's dramatic impact (+35 knowledge points), only 8% of physicians have received it, revealing both the problem and solution. Board certification and formal training emerged as key predictors of competency, while experience alone proved insufficient. Junior physicians, MBBS-only practitioners, and those with limited exposure to travel medicine require urgent intervention. Specific gaps in malaria prophylaxis, air travel counseling, and risk assessment provide clear educational targets.

For a city gateway to millions of pilgrims and expanding tourism under Vision 2030, systematic travel medicine education is imperative. The evidence suggests a clear path forward: expand access to formal training, implement clinical decision support, and establish certification pathways. This investment transcends professional development-it is essential for epidemic preparedness and global health security.

## Appendices

Study questionnaire

Knowledge, Attitudes, and Practices Regarding Travel Medicine Among Primary Healthcare Physicians in Jeddah

Section A: Demographic Information

Age: _______ years

Gender:

Male

Female

Nationality:

Saudi

Non-Saudi

Healthcare Affiliation:

Jeddah First Health Cluster

Jeddah Second Health Cluster

King Fahad Armed Forces Hospital

National Guard Health Affairs

King Abdulaziz University Hospital

Other

Years of Clinical Experience:

1-2 years

3-5 years

6-8 years

More than 8 years

Highest Degree Obtained:

MBBS/MD

Master's Degree (MPH, MSc, or equivalent)

Board Certified (Saudi Board, Arab Board, or equivalent)

Other Postgraduate

Section B: Knowledge Domain

K1. How many patients with travel-related inquiries do you see per month?

Less than 5

5-10

More than 10

K2. Have you received formal training in travel medicine?

No

Yes

For questions K3-K11, please rate on a scale of 1 to 5: (1 = Not at all aware/familiar/confident, 5 = Very aware/familiar/confident)

K3. To what extent are you aware that travel can be associated with specific morbidity and mortality? 1 - 2 - 3 - 4 - 5

K4. How familiar are you with the key factors to assess during a pre-travel health evaluation? 1 - 2 - 3 - 4 - 5

K5. How well do you know the current guidelines for vaccinations required for international travelers? 1 - 2 - 3 - 4 - 5

K6. How familiar are you with the medical indications for yellow fever vaccination in travelers? 1 - 2 - 3 - 4 - 5

K7. Are you familiar with places where meningococcal vaccination is required, like the African meningitis belt? 1 - 2 - 3 - 4 - 5

K8. How confident are you in prescribing vaccinations required for travelers attending the Hajj pilgrimage? 1 - 2 - 3 - 4 - 5

K9. How confident are you in advising patients on safe food and water hygiene during travel? 1 - 2 - 3 - 4 - 5

K10. To what extent are you aware of the guidelines for Stand-by Emergency Treatment (SBET) of malaria for travelers? 1 - 2 - 3 - 4 - 5

K11. How well do you understand the specific public health challenges related to large-scale mass gatherings like Hajj and Umrah? 1 - 2 - 3 - 4 - 5

Section C: Attitude Domain

For questions A1, A2, and A4, please rate on a scale of 1 to 5: (1 = Strongly disagree, 5 = Strongly agree)

A1. A practicing physician is responsible for providing information on travel medicine to a traveler. 1 - 2 - 3 - 4 - 5

A2. Basic knowledge of travel medicine is part of a modern physician's competence. 1 - 2 - 3 - 4 - 5

A3. Do you believe travel medicine knowledge differs significantly from general medicine, infectious disease, and tropical medicine?

No

Not sure

Yes

A4. There should be a training program on travel medicine for staff at your health center. 1 - 2 - 3 - 4 - 5

A5. Are you interested in participating in a short course on travel medicine at your health center?

No

Not sure

Yes

Section D: Practice Domain

For questions P1-P5, please indicate frequency over the last two years: (1 = Never, 2 = Rarely, 3 = Sometimes, 4 = Often)

P1. How often have you conducted a pre-travel assessment for a traveler? 1 - 2 - 3 - 4

P2. How often have you given pre-travel advice to a person planning to attend a mass gathering (e.g., Hajj)? 1 - 2 - 3 - 4

P3. How often have you advised malaria chemoprophylaxis to a person traveling to a malaria-endemic region? 1 - 2 - 3 - 4

P4. How often have you advised prophylaxis for travelers' diarrhea? 1 - 2 - 3 - 4

P5. How often have you given pre-travel advice specifically for air travel? 1 - 2 - 3 - 4
